# Improvement of Resident Scholarship in an Internal Medicine Training Program

**DOI:** 10.1007/s11606-021-06887-2

**Published:** 2021-06-18

**Authors:** Elizabeth R. Doman, Michael S. Abdo, Dacia S. K. Boyce, Daniel H. Desmond, Joseph L. Roswarski, David C. Hostler

**Affiliations:** 1 Department of Medicine, Tripler Army Medical Center, 1 Jarrett White Road, Honolulu, HI 96859 USA; 2 Katterbach Kaserne, Ansbach, Germany; 3 Division of Hematology/Oncology, Department of Medicine, Walter Reed National Naval Medical Center, Bethesda, MD USA; 4 Womack Army Medical Center, Ft. Bragg, NC USA

## INTRODUCTION

The Accreditation Council for Graduate Medical Education (ACGME) stipulates that residents must participate in scholarship, and programs must provide curricula to accomplish this. Residents understand that they should participate in scholarly activity, but are often dissatisfied with their program’s approach.^1^ Evidence of effective interventions that lead to tangible scholarship in 3-year residencies is inconsistent. Recent systematic reviews have described initiatives utilized by ACGME programs to increase scholarship.^2,3^ Tripler Army Medical Center (TAMC) Internal Medicine (IM) residency program educates a diverse group of civilian and active duty military residents. We initiated simple, reproducible interventions to improve resident participation in and understanding of the medical research process, based on previously published methods.^2–6^

## METHODS

As a process improvement study, a structural framework was developed for scholarly activity within the TAMC IM residency program. A total of 32–39 IM residents per year participated. The interventions took place at the start of the 2016–2017 academic year. Metrics were collected from July 2016 to June 2019. Metrics from July 2015 to June 2016, before interventions occurred, served as the control. No additional monetary funding was provided.

A Scholarly Activity Council (SAC) was assembled, with a volunteer faculty member serving as Chair (CSAC). The council contained representatives from each IM subspecialty, to act as human resources for projects in their area of expertise. All SAC members were volunteers. The CSAC created a shared access database, including a bulletin of projects needing assistance, deadlines, and a list of project statuses and pending tasks. It was reviewed and updated monthly at a dedicated research conference on the academic schedule, where residents also practiced presentations and discussed projects. Faculty members in attendance at the meeting could give feedback and advice for these presentations or projects. A research curriculum was designed, including monthly lectures on statistical analysis, critical literature appraisal, and guides for manuscript writing. Protected longitudinal research time was added to resident schedules, with 4 weeks of dedicated time per academic year. This time replaced one elective rotation block.

From July 2016 to June 2019, residents were requested to report any new scholarly activity to the Chief of Medical Residents. Metrics collected to evaluate participation included quantity and type of manuscripts published, presentations at local/regional/national conferences, and ongoing or new Institutional Review Board (IRB)–reviewed studies. Abstracts’ or manuscripts’ pending acceptance and in-house scholarship, such as morning report or grand rounds, were excluded.

## RESULTS

From July 2015 to June 2016, 4 manuscripts and 2 book chapters were published, and 22 presentations were given. This represented the program’s baseline scholarly activity. During the 2016–2017 academic year, presentations increased to 45 (Table 1). This increase remained stable, with overall 186% growth over 3 years. Accepted peer-reviewed manuscripts surged to 8, and then 14. This represents a 350% growth from the initiation of interventions. In June 2016, an additional 8 manuscripts were pending acceptance. No active IRB research protocols existed in 2015, but rose to 4 by 2019.

**Table 1:** **Scholarly Activities by Type, July 2015 to June 2019. Quantities of Posters, Presentations, Book Chapters, Manuscripts Accepted for Publication, and Active IRB Protocols, in Chronological Order. 2015–2016 Refers to the Pre-intervention Academic Year, with the Three Subsequent Columns Representing Academic Years During which Interventions Were Active**

	Pre-interventions	Intervention period		
Academic year	2015–2016	2016–2017	2017–2018	2018–2019
Posters/presentations	22	45	39	41
Book chapters	2	0	1	2
Accepted manuscripts	4	3	8	14
Active IRB protocols	0	3	3	4
Number residents as first author	4	3	8	14
Number of residents per year	32	34	39	39

## DISCUSSION

Scholarship increased within the TAMC IM residency program after instituting reproducible, evidenced-based interventions^2–6^ requiring minimal resources. Overall, the interventions described established a collaborative environment between faculty and residents (Table 2).

**Table 2:** **List of Primary Interventions with Their General Purpose, and Subjective Effects Observed by the Authors Over the Course of the Intervention Period**

Interventions	Purpose	Outcome
Scholarly Activity Council (SAC)	Assemble faculty to act as mentors Able to assist with scholarship in their subspecialties Create a standardized review process for manuscript submissions	Encouraged faculty and resident cooperation and idea sharing Facilitated scholarship dedication and completion Abstract/manuscript submissions were reviewed by multiple faculty members
Chair of Scholarly Activity (CSAC)	Coordinate assembly of the SAC Lead monthly research meeting Rotation director for research block Write and institute research curriculum	Changed the culture of the training program Held individuals accountable Fostered professional relationships and promoted academic growth
Comprehensive shared scholarship database	Track new/ongoing projects and pending tasks Introduce residents without experience to process of scholarly activity in low-risk, public forum Give residents opportunities to join projects	Changed the culture of the training program Made scholarship more tangible and accessible for trainees Encouraged mutual accountability for project tasks
Monthly research meeting	Remind participants of deadlines for projects Discuss and address barriers to project progression Allow residents to practice presentations	Changed the culture of the training program Encouraged mutual accountability
Scholarship curriculum	Educate residents in scholarship, including statistical analysis, critical literature appraisal Instruct residents in effective manuscript writing by utilizing multiple staff with publication experience, specific journals’ author instructions, standardized templates (i.e., cover letters, quality improvement fishbone designs, etc.)	Improved scholarship quality and quantity Improved resident understanding of commonly used statistical methods (correlation, comparison of means, and regression), study design, study population and size, bias, applicability to clinical practice, and clarity of original data presentation
Protected research time	Grant residents 4 weeks per academic year dedicated to scholarship	Increased productivity Allowed residents to focus on scholarship efforts Enabled residents to maintain purpose and direction in long-term projects

Other programs have utilized similar interventions to improve scholarly activity.^2–6^ Among primary care specialties, lack of mentorship and protected time to complete scholarship impacts their ability to fulfill the requirement.^4^ Other factors, e.g., prior research experience and desire for fellowship training, may also affect scholarship.^1–4,6^ Our project presents a standardized, literature-derived approach that is transferable across ACGME programs.

Limitations include a small sample size and limited pre-intervention data. It is difficult to quantify which aspects of the multi-pronged interventions were most successful, and to what degree the culture change, signaled by an overt focus on scholarship, augmented the individual interventions. We posit that the cumulative effect of the interventions outweighed the sum of their parts.

By developing a stable research environment with designated mentors, we increased our production of peer-reviewed publications. Key to the success of the program was the designation of the CSAC, who was empowered to make substantial, meaningful changes. Further work is needed to evaluate how other specialties may benefit from similar interventions, and to refine further the approach to scholarship across ACGME programs.
